# Supplementary material for: Segmented multiblock polyolefin compatibilizers from non-living metathesis chain-shuffling
Source: Nat Commun. 2025 Nov 26;16:10515. doi: 10.1038/s41467-025-65525-1 (PMC12658212; doi:10.1038/s41467-025-65525-1)
Supplement: Supplementary file 1 — Supplementary Information [file 41467_2025_65525_MOESM1_ESM.pdf]

Supplementary Information

Segmented Multiblock Polyolefin Compatibilizers from Non-Living Metathesis Chain-Shuffling

Abhishek Banerjee,[1] Navin Kafle,[1] Walter G. Romano,[1] Harsh Pandya,[1] Shin Horiuchi,[2] Aarushi Srivastava,[1] Fardin Khabaz,[1] Mark D. Foster,[1] Toshikazu Miyoshi,[1] James M. Eagan[1]*

[1] *The School of Polymer Science and Polymer Engineering, The University of Akron, Akron, OH, 44303, United States of America*

[2] *Research Laboratory for Adhesion and Interfacial Phenomena, Nanomaterials Research Institute, National Institute of Advanced Industrial Science and Technology, Tsukuba, Ibaraki 305-8565, Japan*

Nuclear magnetic resonance spectra 2
High temperature size exclusion chromatography 16
Solid state NMR proton broadline 18
Wide angle x-ray diffraction 18
Uniaxial tensile tests 19
Transmission electron microscopy 20
Unsaturated stability study by sol/gel and molar mass 21
Hydrogenation of multiblock 22
Additional mechanical properties of blends 23
Effect of telechelic functionality on degree of polymerization 24
Scanning electron microscopy and quantification of average droplet diameter 25

Nuclear magnetic resonance spectra

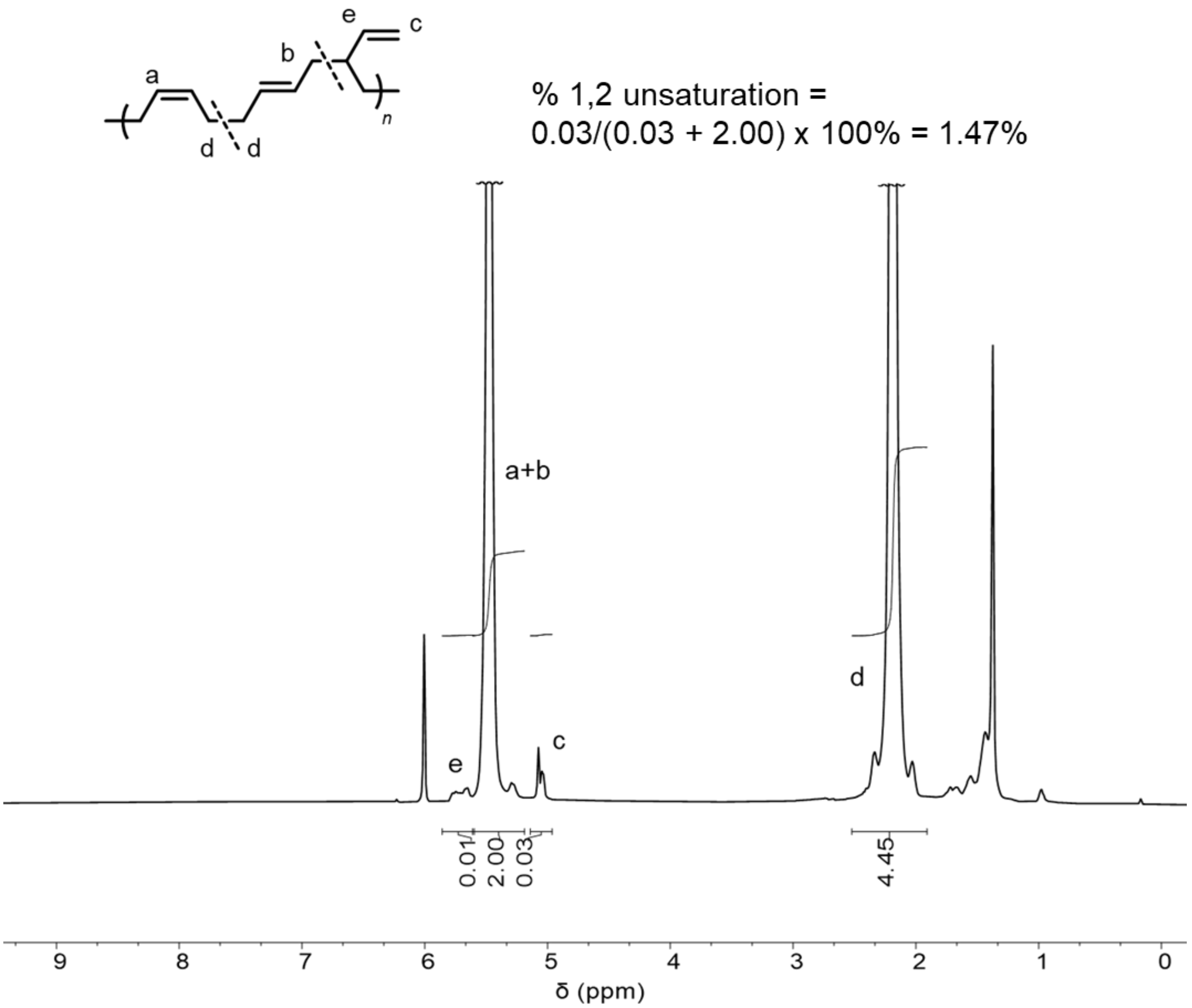

Figure S1. ^{1}H NMR ($C_2D_2Cl_4$, 400 MHz, 125 °C for comparison purposes, 270° pulse width and 6 second relaxation) spectrum of starting polybutadiene materials.

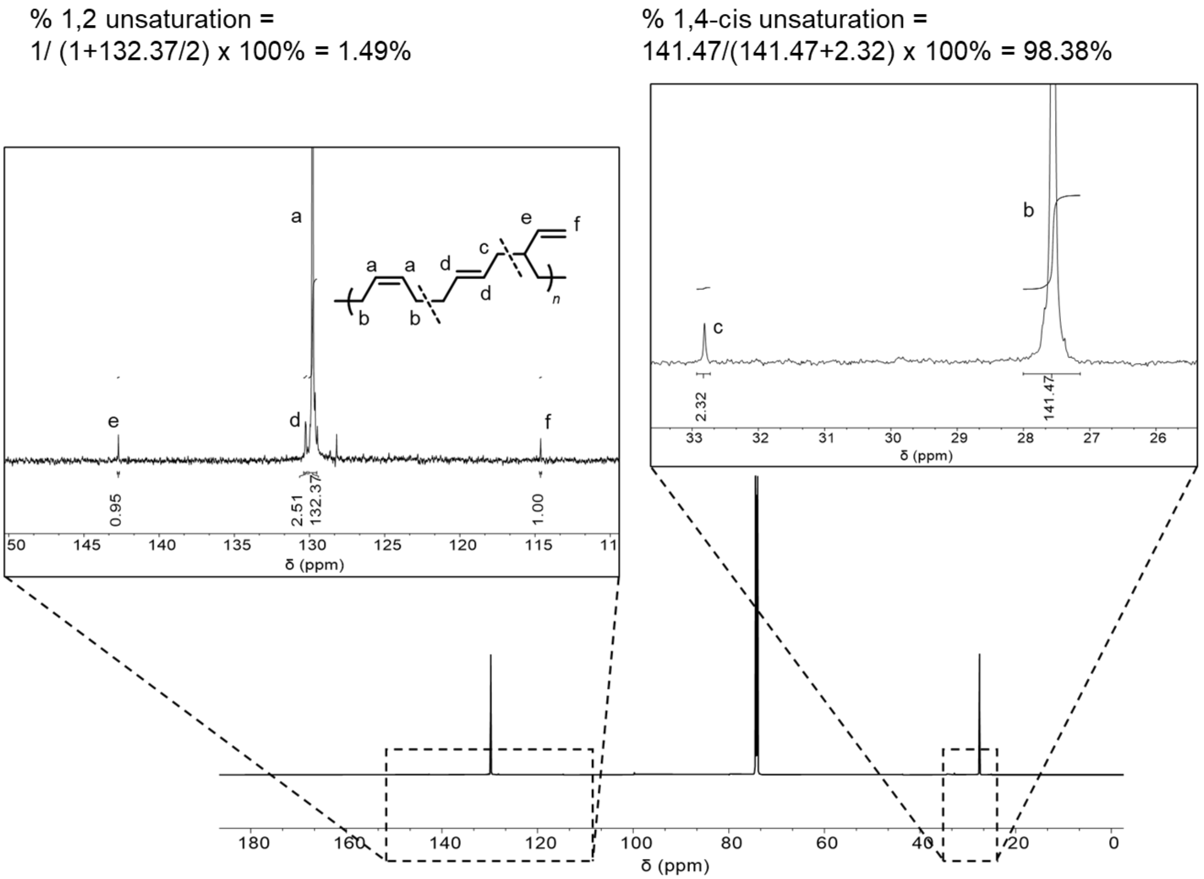

Figure S2. ^{13}C NMR ($C_2D_2Cl_4$, 125 MHz, 25 °C, 270° pulse width and 6.5 second relaxation, containing 0.025M $Cr(acac)_3$ as a relaxation agent) spectrum of starting polybutadiene materials.

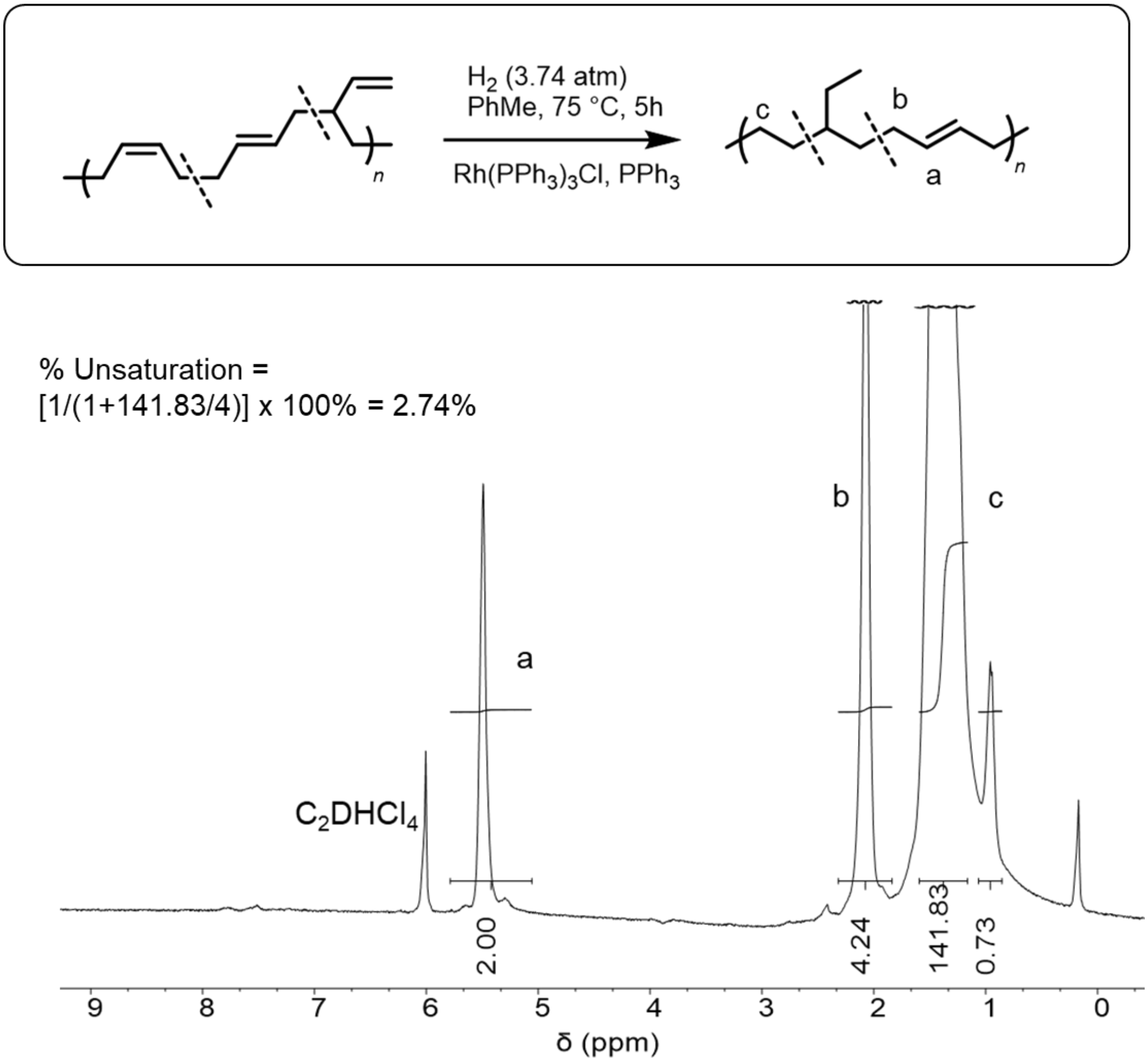

Figure S3. ^{1}H NMR (C_2DHCl_4, 400 MHz, 125 °C, 270° pulse width and 6 second relaxation) spectrum of unsaturated polyethylene (uPE).

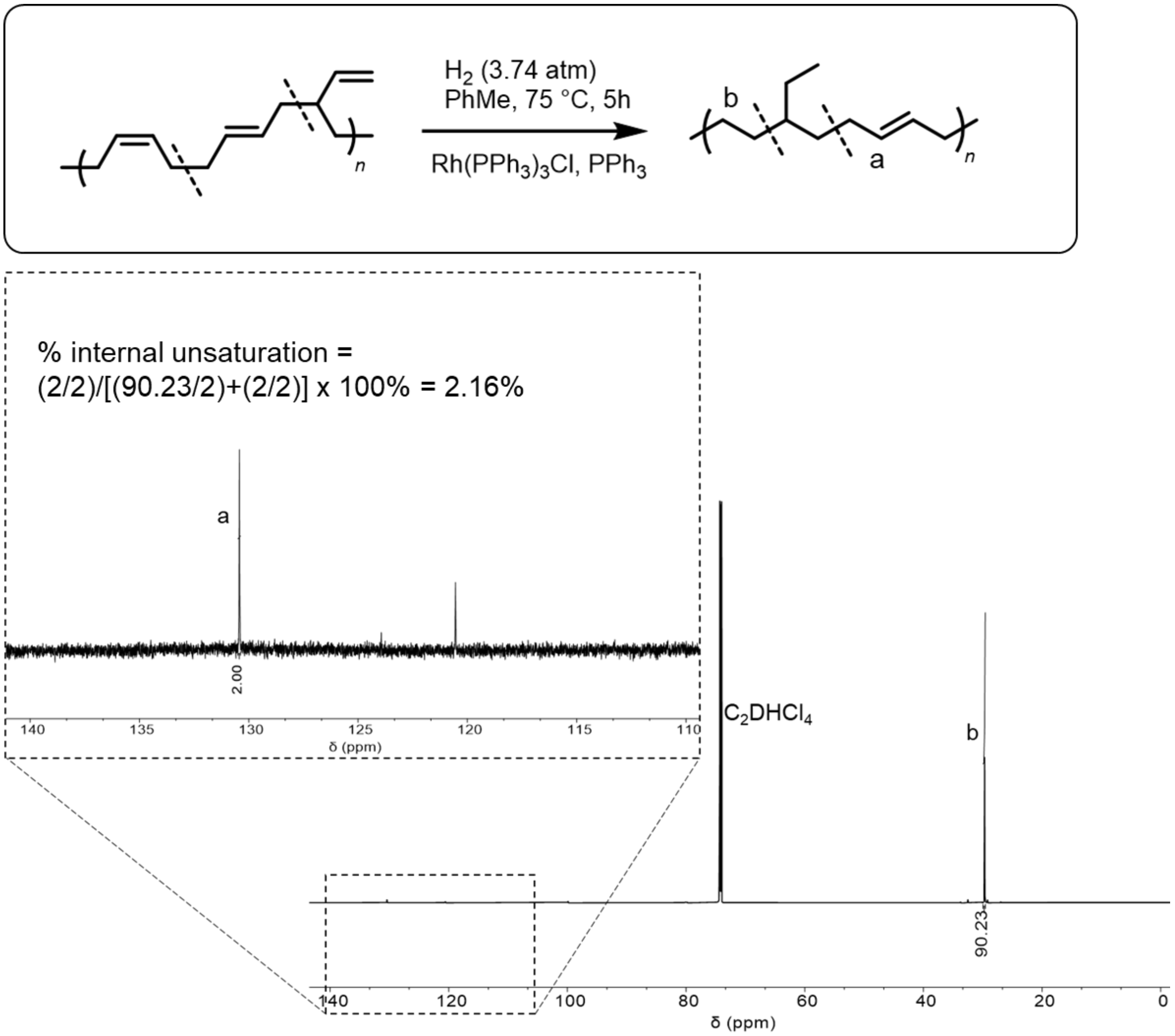

Figure S4. ^{13}C NMR ($C_2D_2Cl_4$, 187.5 MHz, 125 °C, 270° pulse width and 6.5 second relaxation, containing 0.025M $Cr(acac)_3$ as a relaxation agent) spectrum of unsaturated polyethylene (uPE).

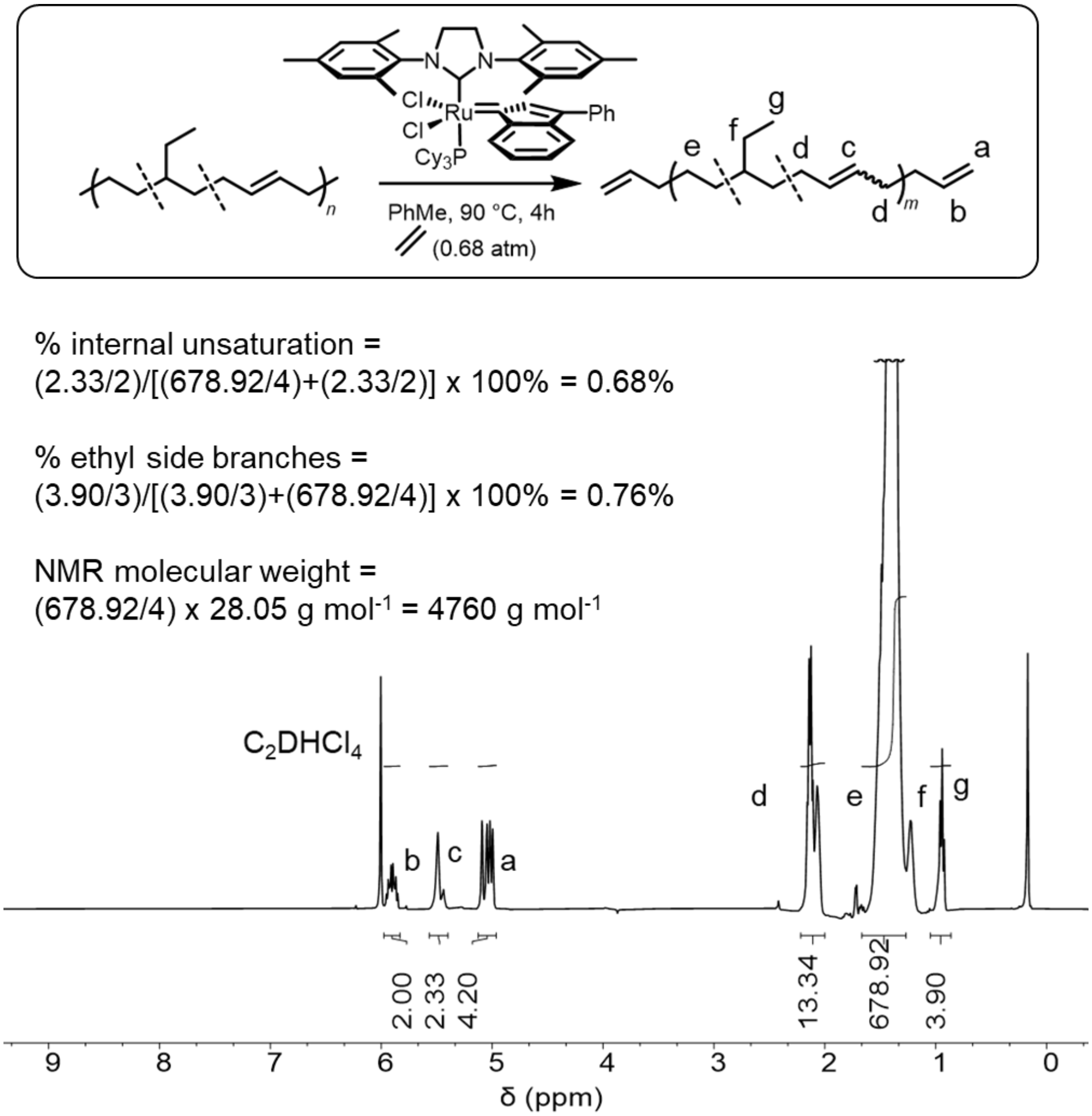

Figure S5. ^{1}H NMR (C_2DHCl_4, 400 MHz, 125 °C, 270° pulse width and 6 second relaxation) spectrum of telechelic polyethylene.

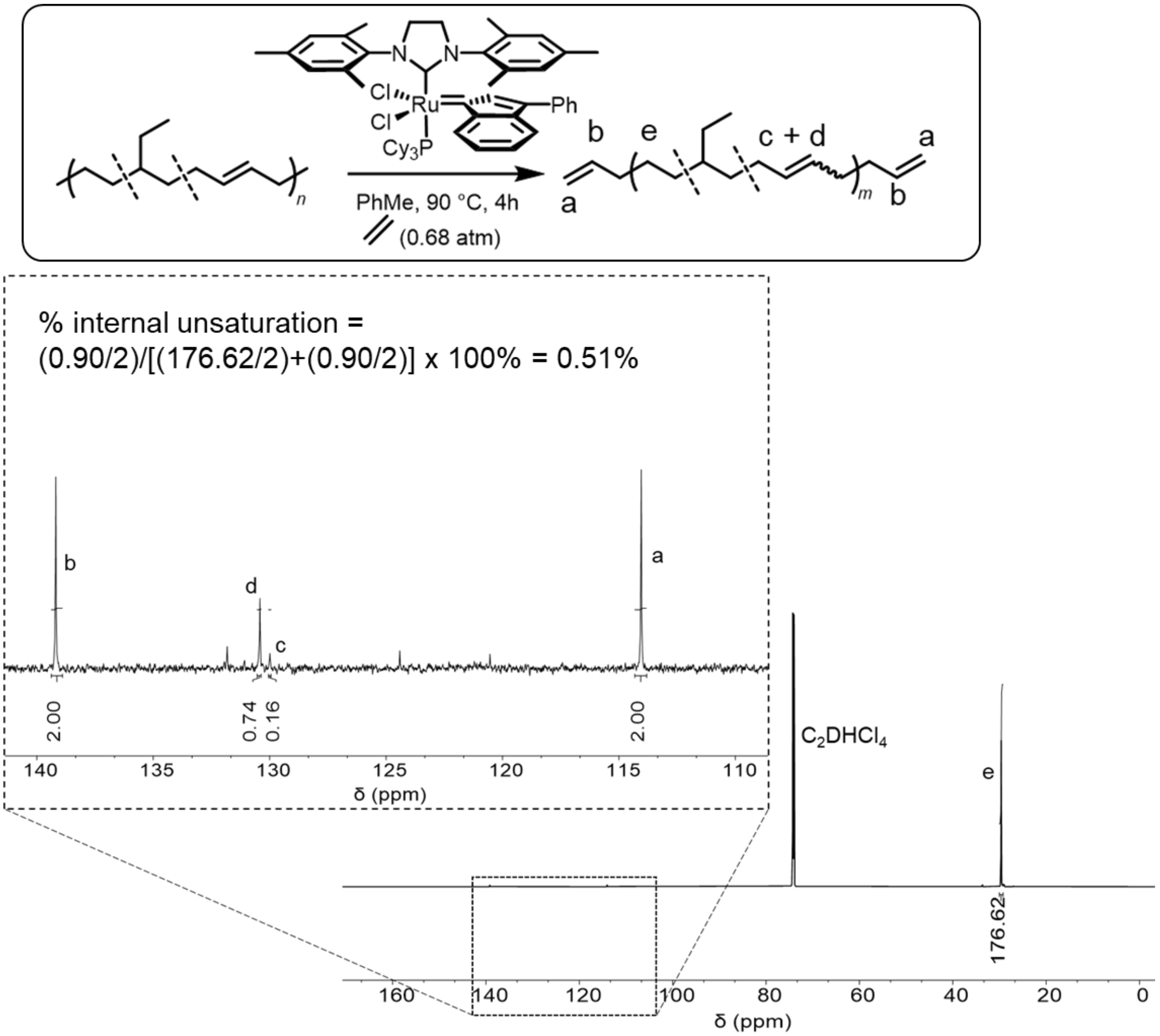

Figure S6. ^{13}C NMR ($C_2D_2Cl_4$, 187.5 MHz, 125 °C, 270° pulse width and 6.5 second relaxation, containing 0.025M $Cr(acac)_3$ as a relaxation agent) spectrum of telechelic polyethylene.

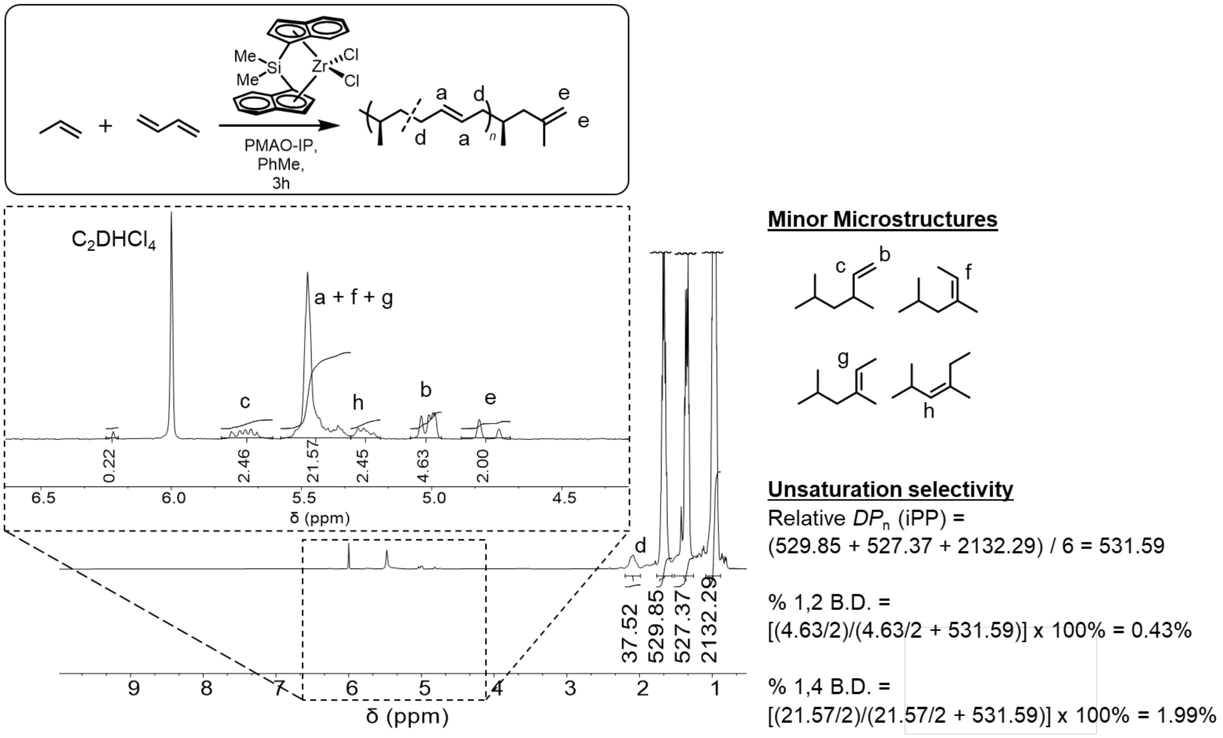

Figure S7. ^{1}H NMR ($C_2D_2Cl_4$, 400 MHz, 125 °C, 270° pulse width and 6 second relaxation) spectrum of unsaturated polypropylene (uPP).

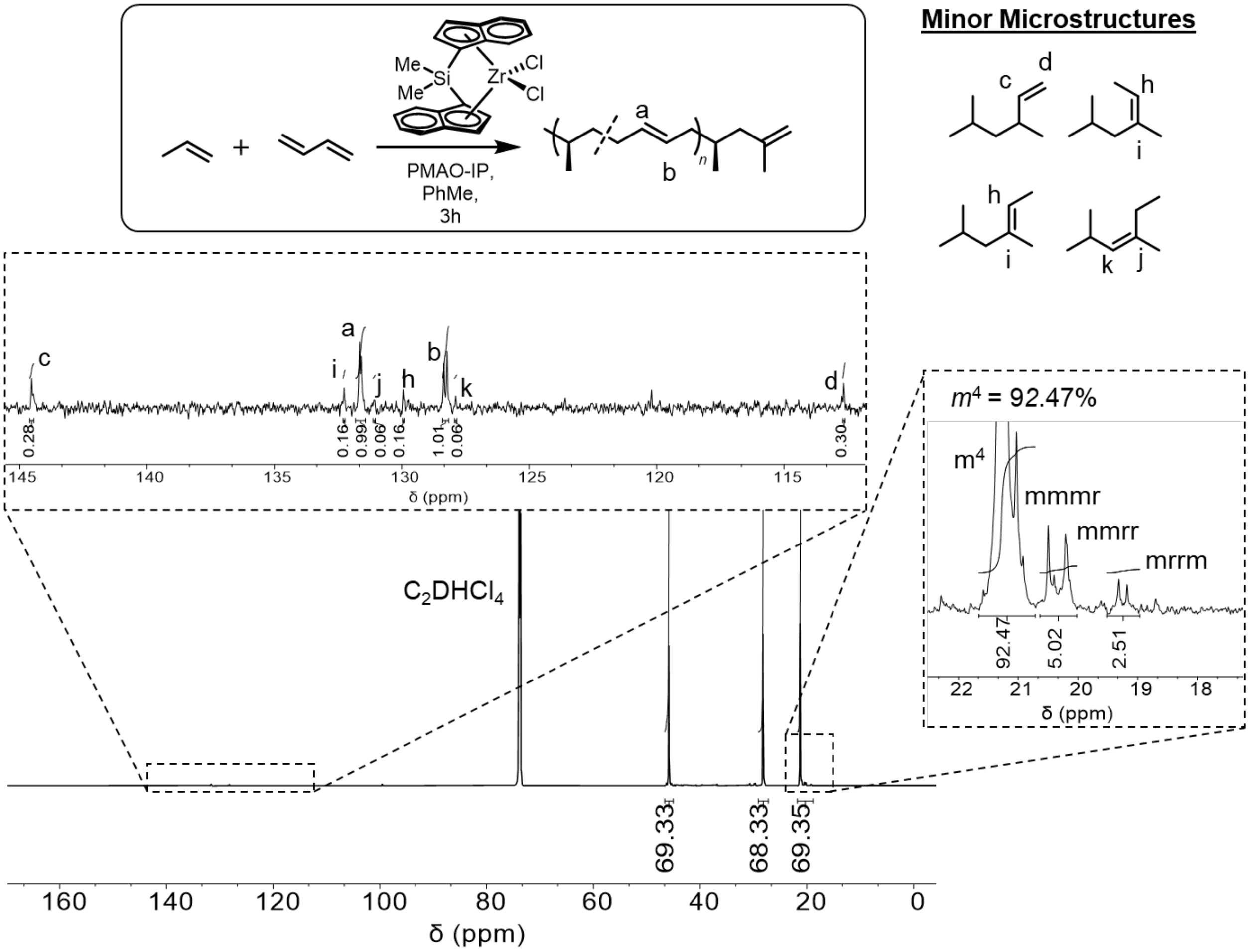

Figure S8. ^{13}C NMR ($C_2D_2Cl_4$, 187.5, 125 °C, 270° pulse width and 6.5 second relaxation, and 0.025M of $Cr(acac)_3$ as a relaxation agent) spectrum of unsaturated polypropylene (uPP).

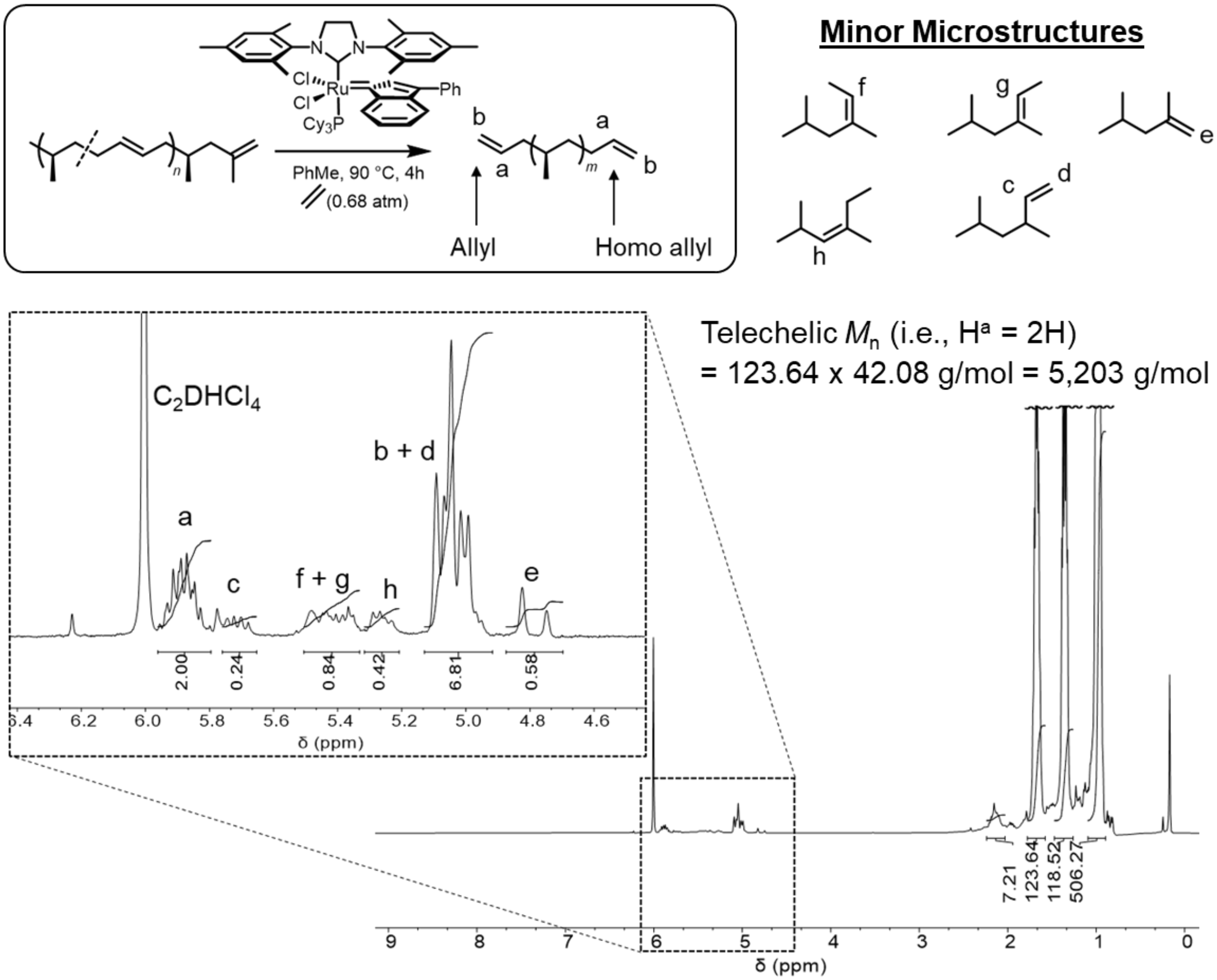

Figure S9. ^{1}H NMR ($C_2D_2Cl_4$, 400 MHz, 125 °C, 270° pulse width and 6 second relaxation) spectrum of telechelic polypropylene.

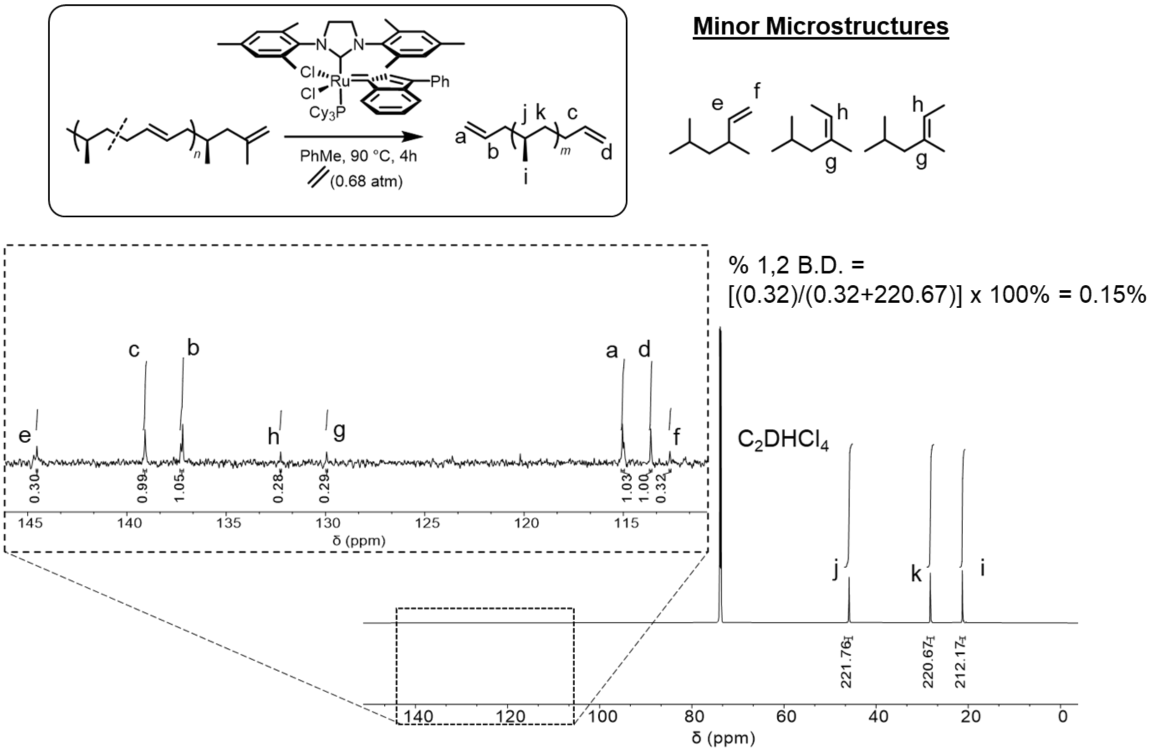

Figure S10. ^{13}C NMR ($C_2D_2Cl_4$, 187.5 MHz, 125 °C, 270° pulse width and 6.5 second relaxation and 0.025M $Cr(acac)_3$ as a relaxation agent) spectrum of telechelic polypropylene.

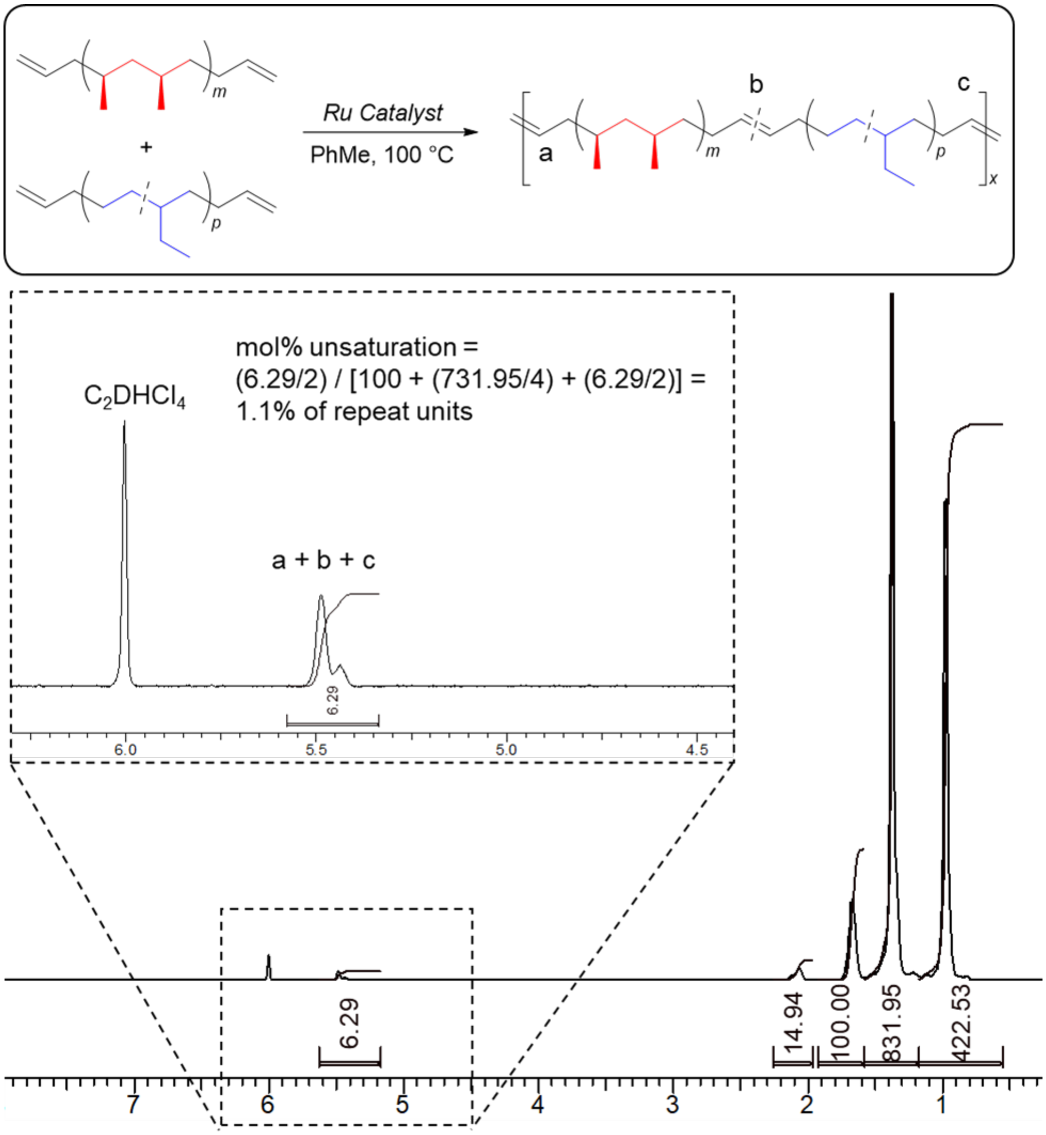

Figure S11. ^{1}H NMR ($C_2D_2Cl_4$, 400 MHz, 125 °C, 270° pulse width and 6 second relaxation) spectrum of multiblock [iPP-*b*-LLDPE]$_x$ from metathesis polymerization.

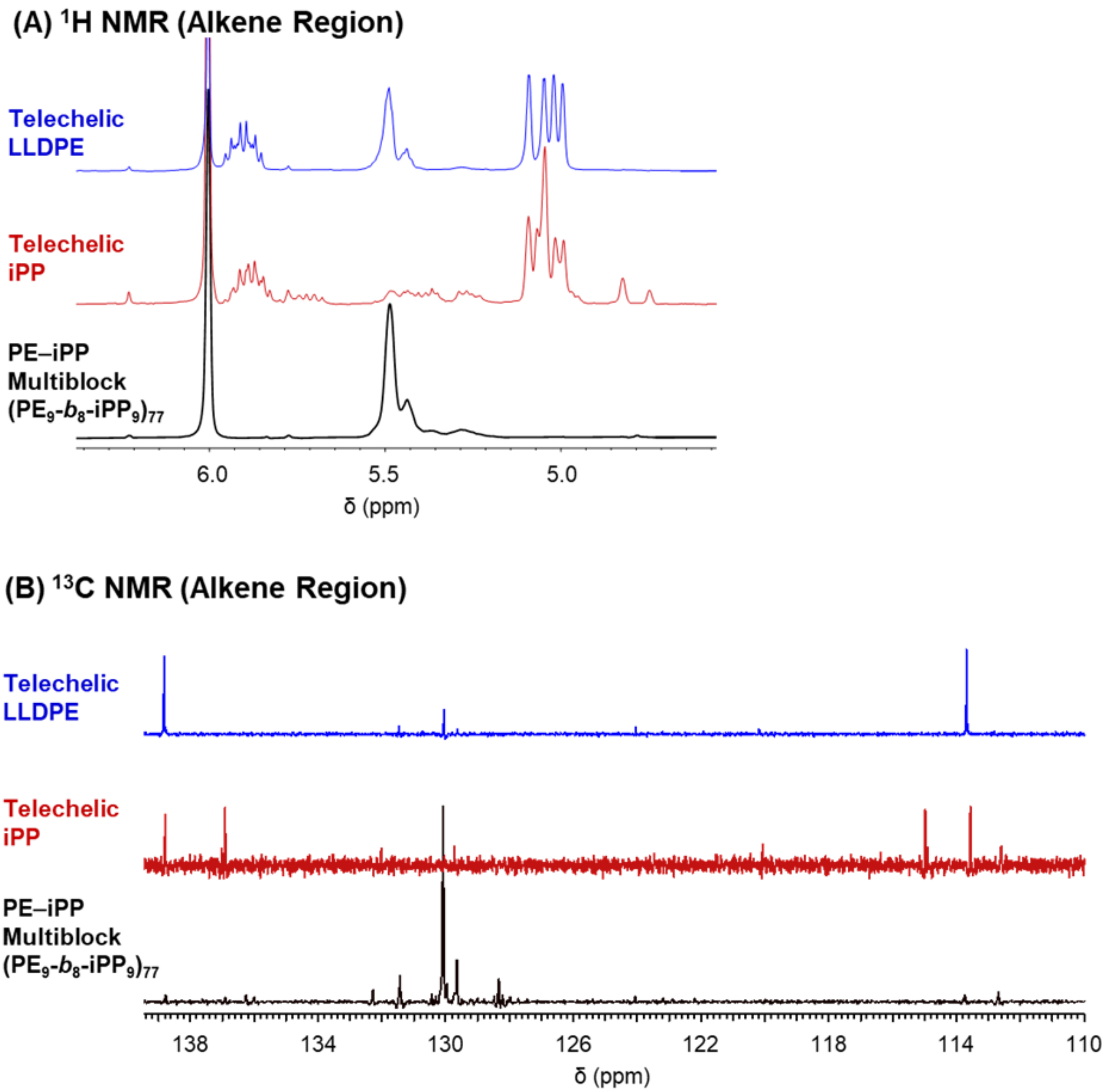

Figure S12. Comparison of precursors and multiblock products by ^{1}H NMR (400 MHz) and ^{13}C NMR ($C_2D_2Cl_4$, 187.5 MHz, 125 °C).

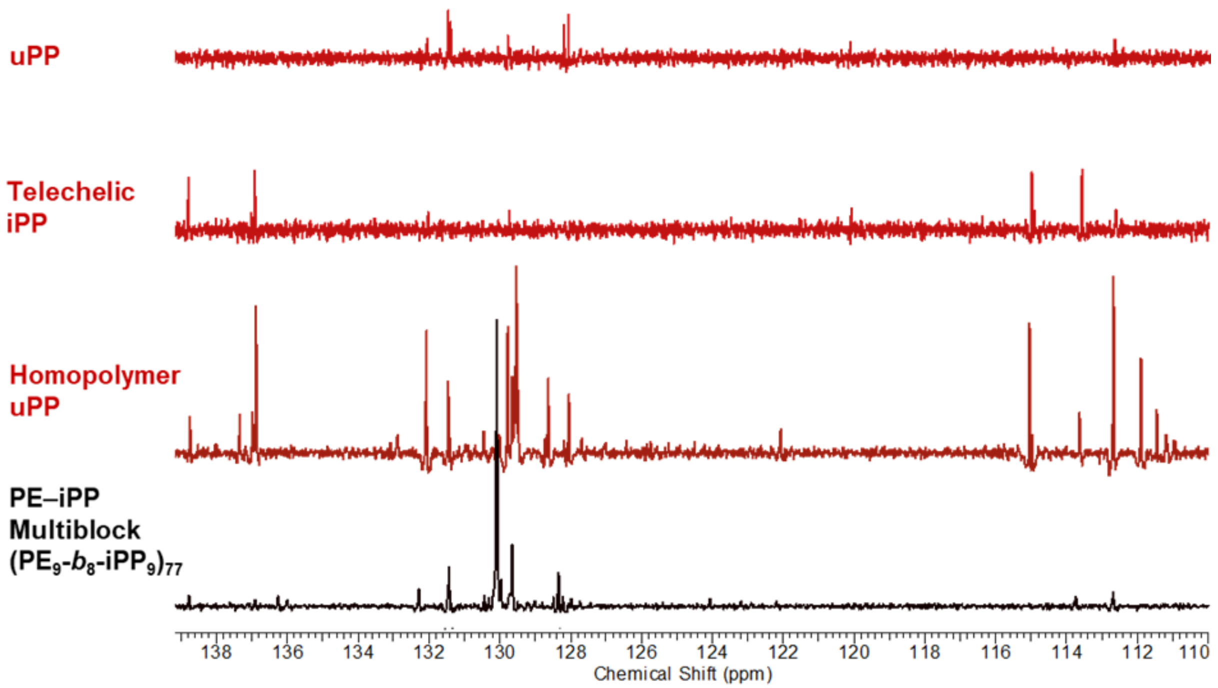

Figure S13. Comparison of uPP, telechelic PP, homopolymerization of telechelic PP, and multiblock products by ^{13}C NMR ($C_2D_2Cl_4$, 187.5 MHz, 125 °C).

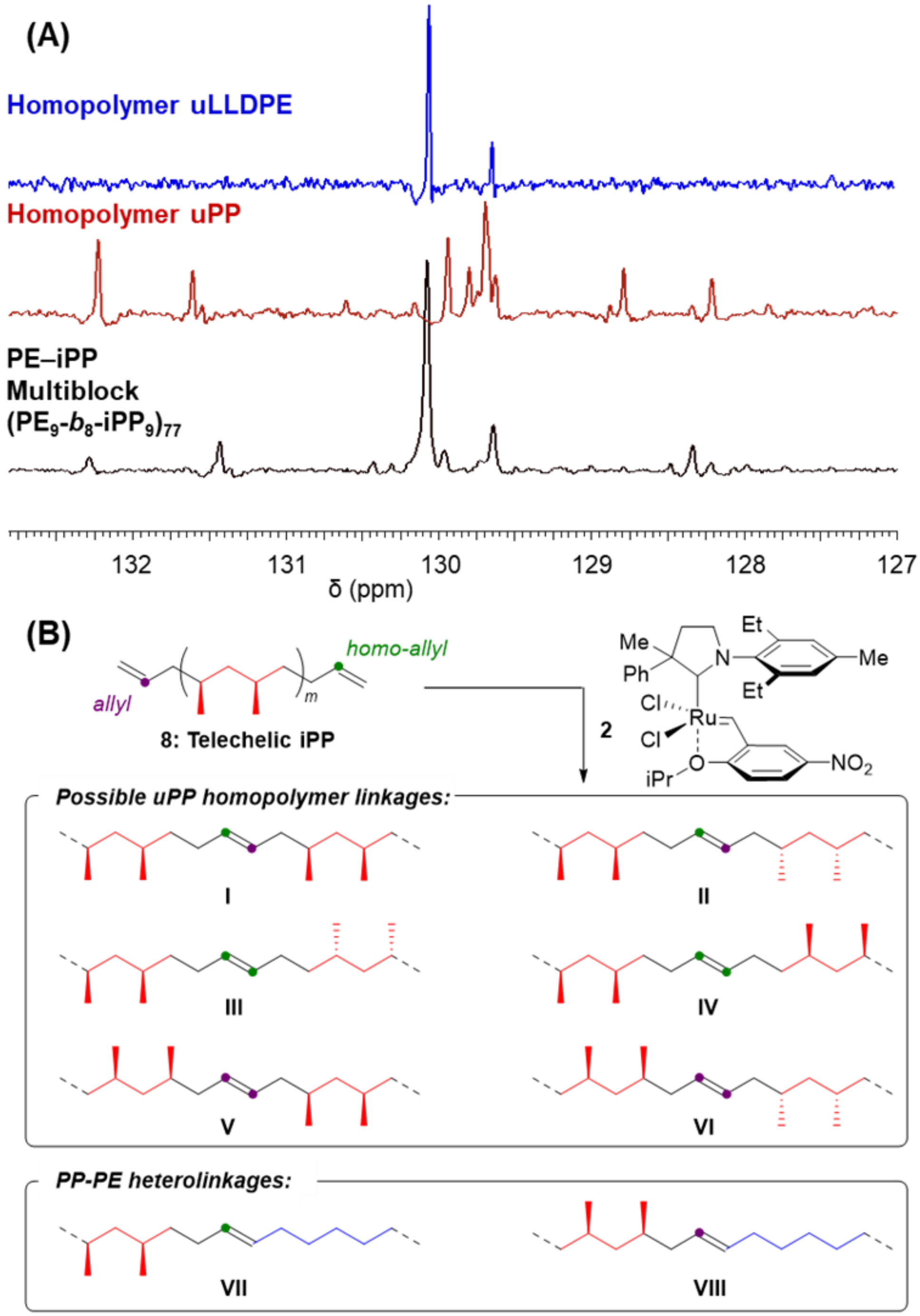

Figure S14. (A) Comparison of products from the homopolymerization of telechelic PP and telechelic PE with the multiblock copolymers, and (B) the rationalized structures from PP homopolymerization (I–VI) and heteropolymerization (VII, VIII) that explain the disappearance of discrete signals in the ^{13}C NMR ($C_2D_2Cl_4$, 187.5 MHz, 125 °C).

High temperature size exclusion chromatography

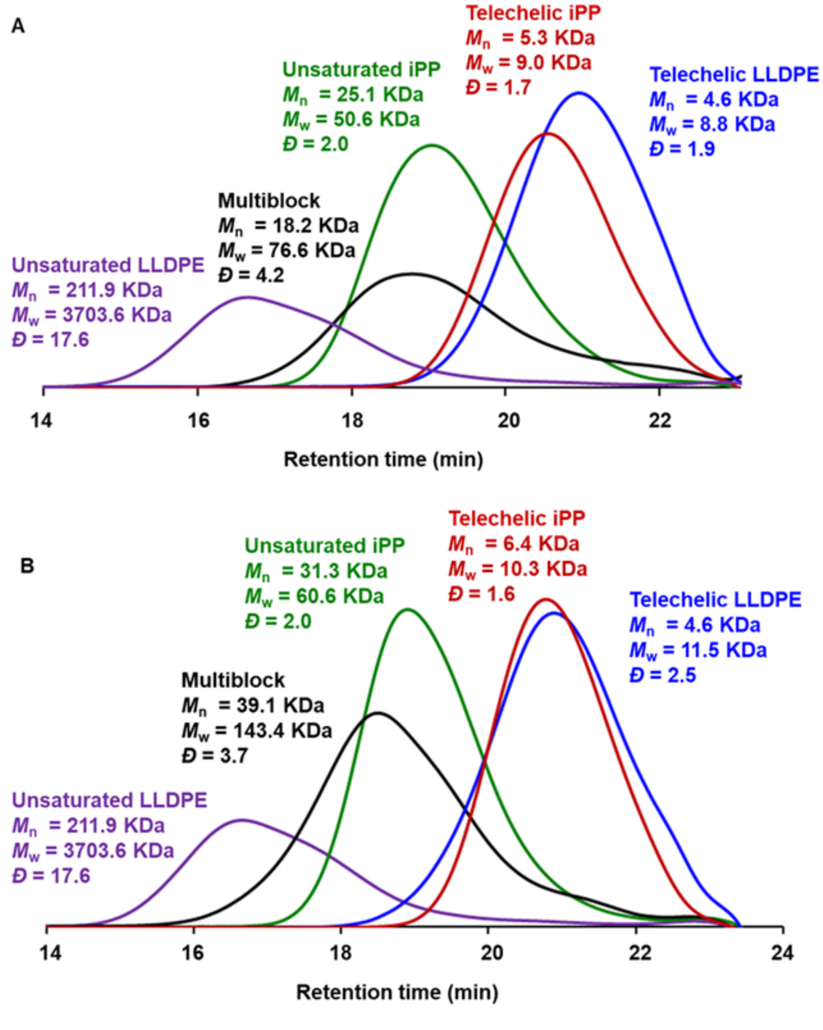

Figure S15. Size exclusion chromatograms of multiblock copolymers and their precursors (1,2,4-trichlorobenzene, 140 °C, and elution rates of 1 mL/min).

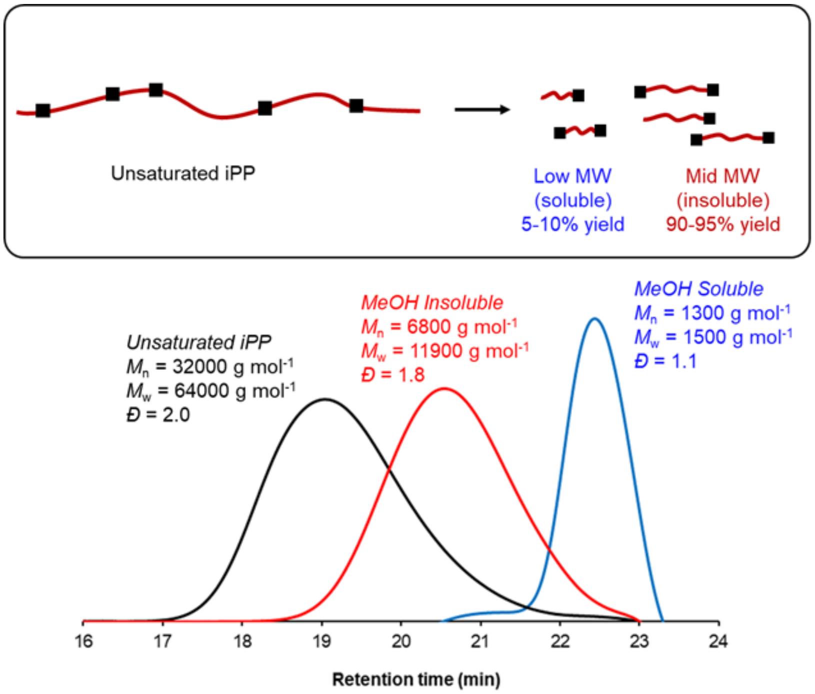

Figure S16. Size exclusion chromatograms of unsaturated iPP and cleavage into low molecular weight telechelics containing localized unsaturated units and therefore low molecular weight methanol soluble fractions in 5–10% yield, depending on the uPP precursor.

Solid state NMR proton broadline

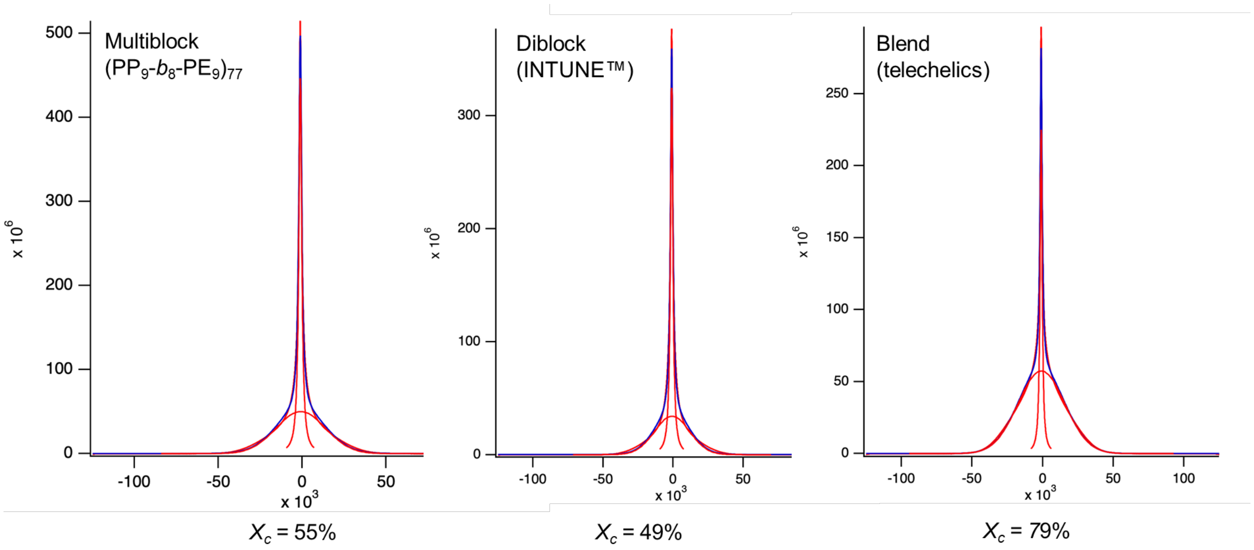

Figure S17. ^{1}H broadline ssNMR spectra for multiblock copolymer, diblock copolymer, and a blend (1:1 wt. ratio) of telechelic polymers.

Wide angle x-ray diffraction

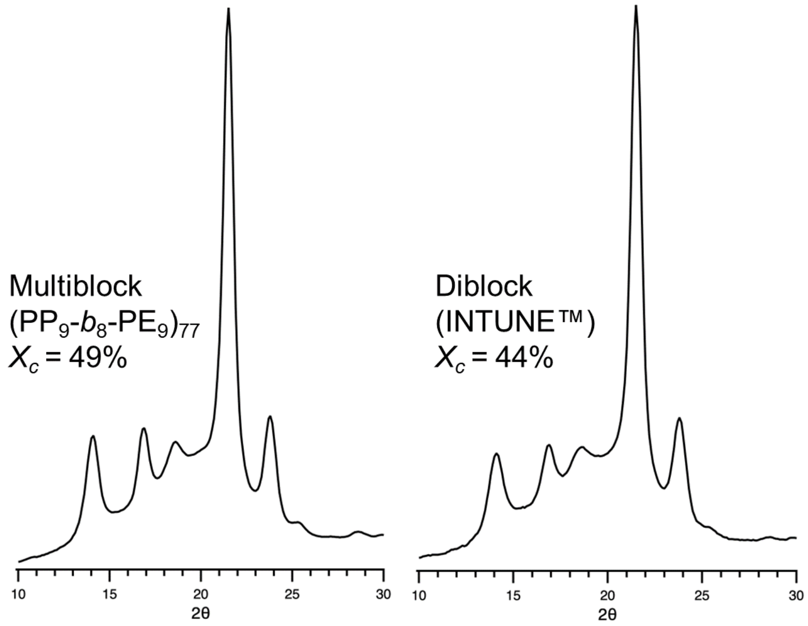

Figure S18. WAXD obtained from a Rigaku Rapid II diffractometer using a 40kV and 30 mA beam monochromatized to Cu Kα radiation, with 30-minute exposure times.

Uniaxial tensile tests

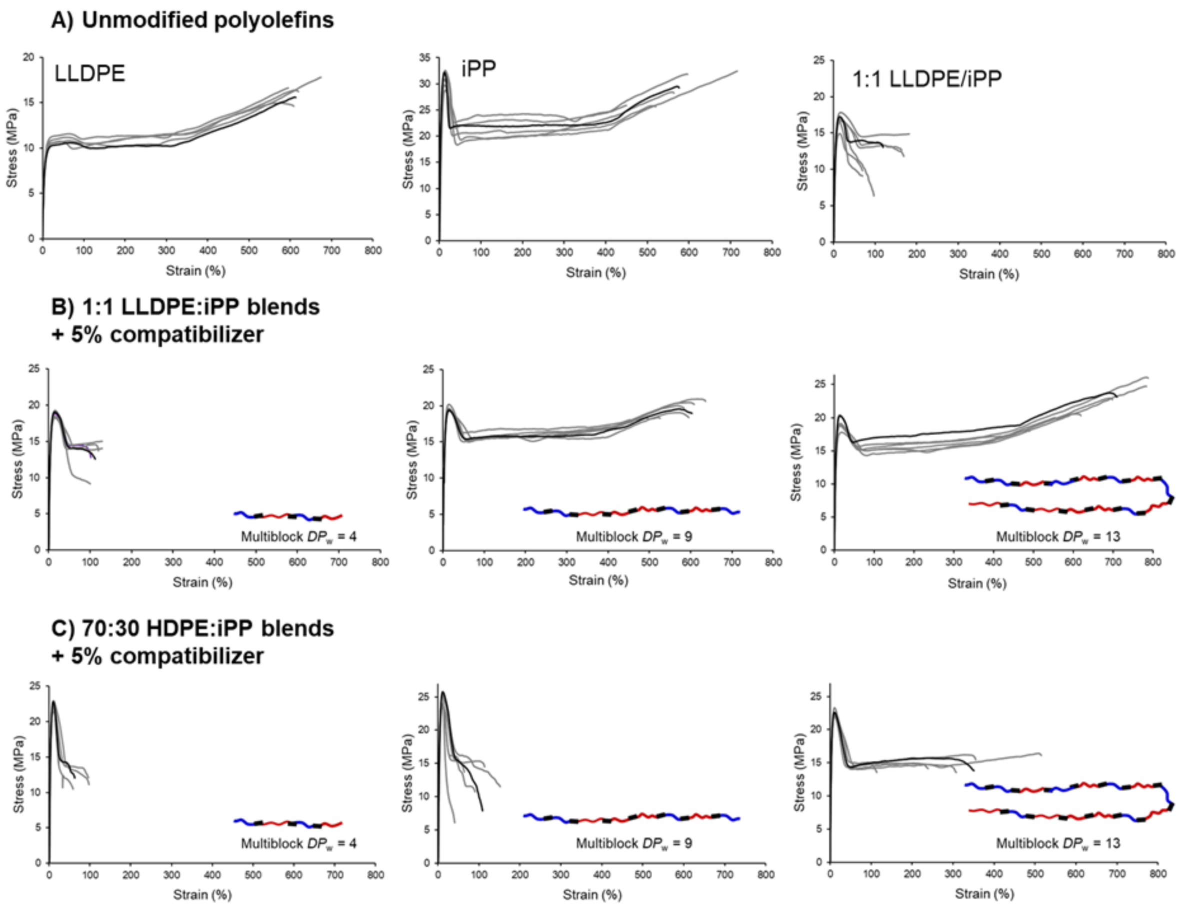

Figure S19. Uniaxial tensile tests of (A) unmodified polyolefins, their blends, and (B) with the addition of multiblock copolymers from Table 1, and (C) when used as HDPE/iPP compatibilizers.

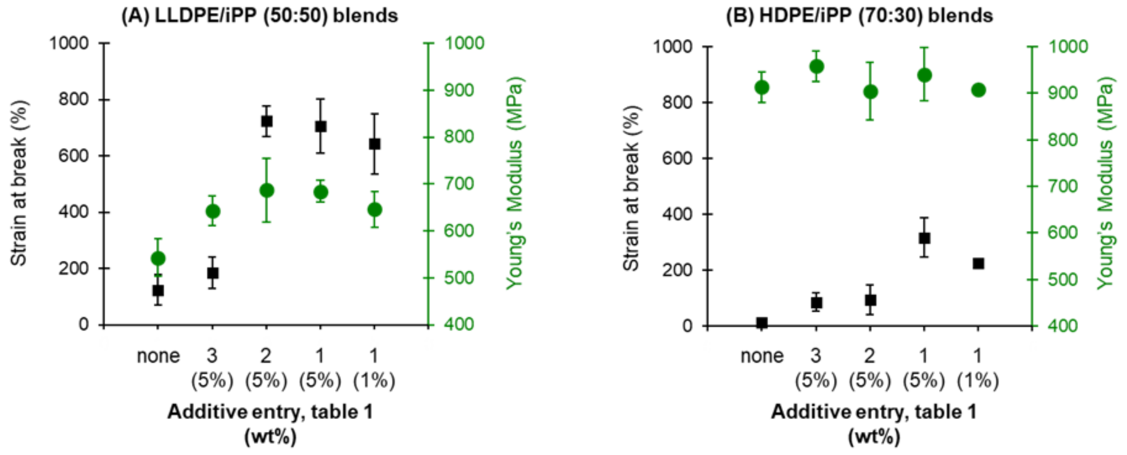

Figure S20. Comparison of Young's modulus and extensibilities, across various copolymer additives and loadings for (A) LLDPE/iPP blends (50:50) and (B) HDPE/iPP blends (70:30). Values presented as the mean value with one standard deviation shown from a minimum of at least 6 test specimens.

Transmission electron microscopy

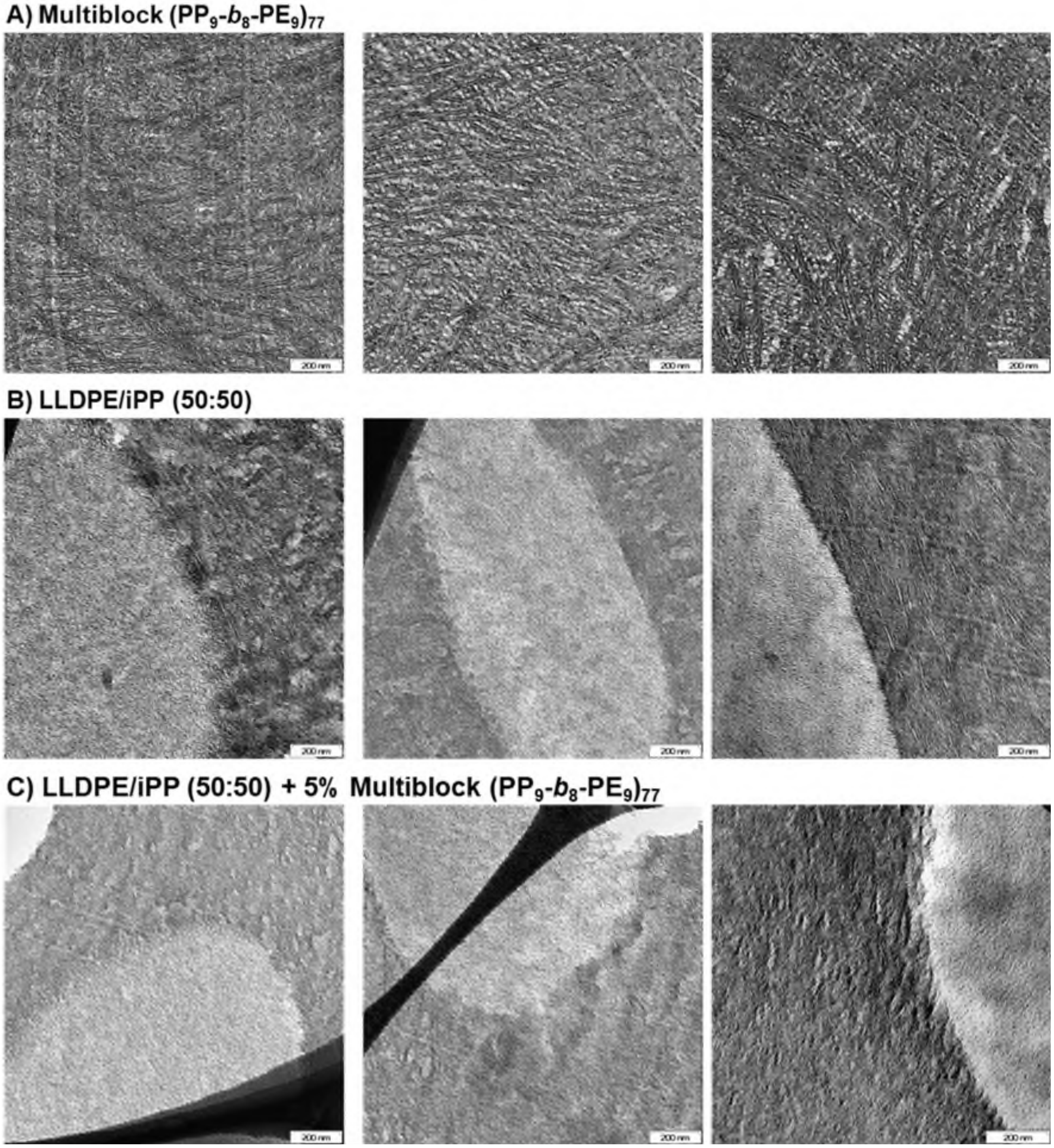

Figure S21. Additional TEM images of multiblock, blends, and compatibilized blends demonstrating the consistency of microscopic observations; cryo-microtomed in liquid nitrogen and stained using RuO_4.

Unsaturated stability study by sol/gel and molar mass

Aging procedures: The synthesized high molecular weight multiblock sample $(PP_{10}\text{-}b_{13}\text{-}PE_{12})_{143}$ was stored in a scintillation vial under air for 20 months after synthesis and reanalyzed as "Air (20 months)". Separately, 250 mg of the material was dissolved in 4 mL of xylenes in a scintillation vial with a magnetic stir bar and heated to 96h with stirring under air. The sample was precipitated in neutral MeOH (20 mL) and decanted to afford an analytical sample labeled "Xylenes, 130 °C, 96h, air". Separately, 500 mg of $(PP_{10}\text{-}b_{13}\text{-}PE_{12})_{143}$ was compression molded into a densified film at 180 °C for 1 minute. This thin film was then fed into the micro-compounder at 185 °C for 7 minutes under continuous nitrogen flow. The heating was turned off, chamber opened, and the melt processed sample removed by spatula to afford analytical sample "Melt, 185 °C, 7m, N_2". Each sample was directly analyzed by high temperature SEC analysis (Figure S22).

Sol/gel measurements: Targeted 200 mg of aged samples were measured into a pre-weighed cellulose thimble and charged into a continuous Soxhlet extractor. 250 mL of toluene were added to a round bottom flask and continuously condensed and extracted through the thimble for 18-22h. The toluene was reduced in volume using a rotovap and then precipitated in neutral methanol, decanted, and dried in a vacuum oven at 60 °C for 24h and weights recorded.

Aging conditions	Crude wt.	Wt.% PhMe soluble	M_w (M_w/M_n)
None (as synthesized)	-	-	143,400 (3.7)
Air (20 months)	200 mg	98%	137,800 (3.2)
Xylenes, 130 °C, 96h, air	202 mg	>99%	149,000 (3.9)
Melt, 185 °C, 7m, N_2	160 mg	96%	151,200 (3.6)

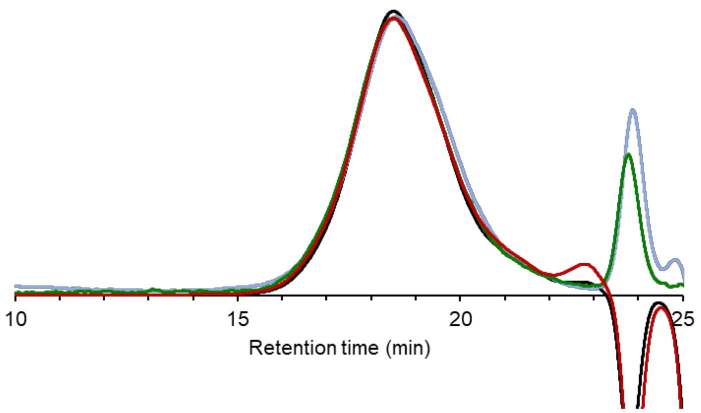

Figure S22. High temperature SEC analysis of aged polyunsaturated multiblocks indicating no significant crosslinking, chain-scission, or degradation. Calibrated against PS standards with Mark–Houwink corrections adjusted for the iPP block between 15–23 minutes; a solvent degassing signal is observed at 24 minutes.

Hydrogenation of multiblock

Procedures: 150 mg of unsaturated multiblock $(PE_{12}\text{-}b_{13}\text{-}iPP_{10})_{143}$ was added into a stainless steel Parr reactor, followed by 50 mg of heterogeneous catalyst (10% Pd/C *or* 5% $Pd/CaCO_3$) *or* 15 mg of homogeneous catalyst $[Ir(cod)(PCy_3)(py)]PF_6$ was added, followed by 5 mL of solvent (PhMe or cyclohexane). The vessel was sealed, purged with nitrogen, and pressurized with hydrogen gas. The system was protected behind a blast-shield and heated to the target temperature for 24h. The reactions were allowed to cool, vented, and allowed to settle. The solution was decanted into methanol (50mL) resulting in a white precipitate that was filtered and dried under vacuum.

For transfer hydrogenation, 150 mg of unsaturated multiblock $(PE_{12}\text{-}b_{13}\text{-}iPP_{10})_{143}$ and 160 mg of *p*-toluenesulfonyl hydrazide (~20 equiv. per alkene) was weighed into a scintillation vial. Then 5 mL of p-xylene was added along with 60 mg of Bu_2NH (to neutralize sulfonic acid side-products) and the mixture heated to 130 °C on a heating block for 24h. The resulting solution was precipitated in 50 mL of methanol, filtered, and dried under vacuum.

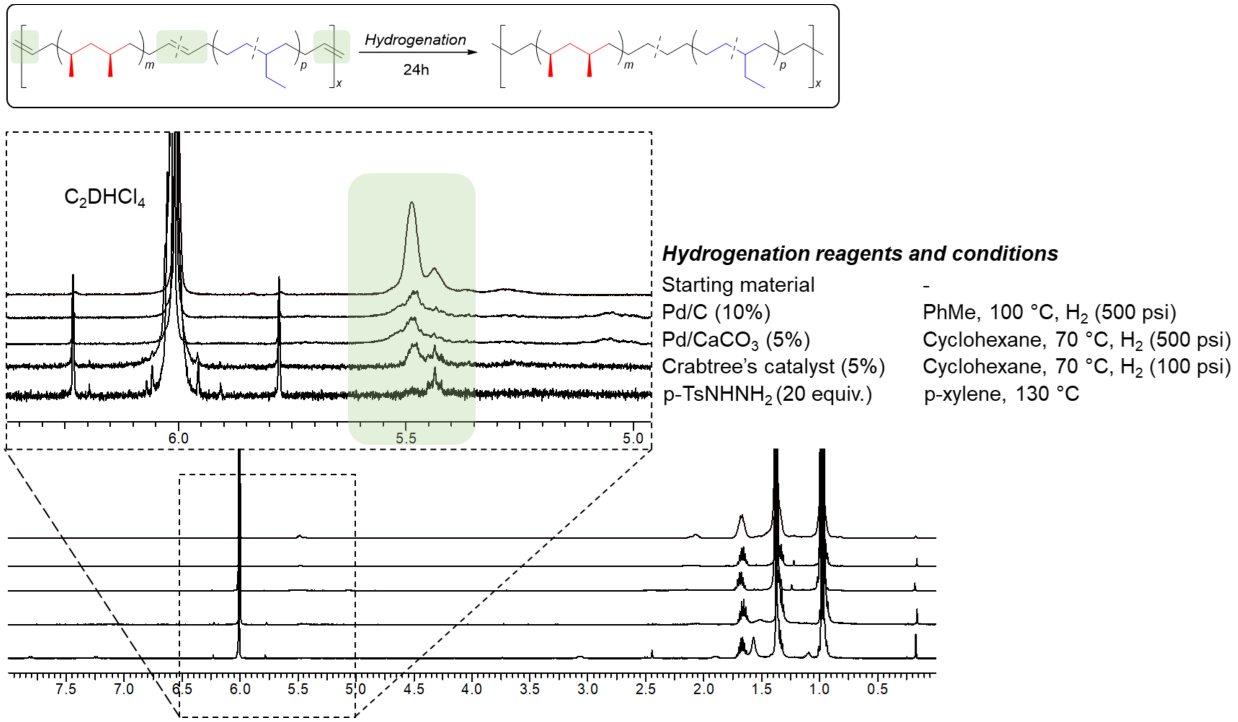

Figure S23. High temperature ^{1}H NMR (CD_2Cl_4, 125 °C, 500 MHz) of products derived from various hydrogenation reactions with $(PE_{12}\text{-}b_{13}\text{-}iPP_{10})_{143}$.

Additional mechanical properties of blends

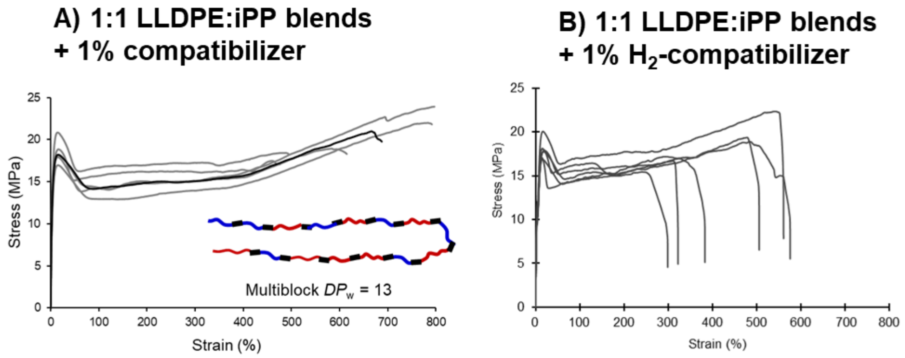

Figure S24. The comparison of $(PE_{12}\text{-}b_{13}\text{-}iPP_{10})_{143}$ compatibilization as the polyunsaturated sample (A) to the results using the same loading (1 wt.%) of hydrogenated compatibilizer from pTsNHNH$_2$ (B).

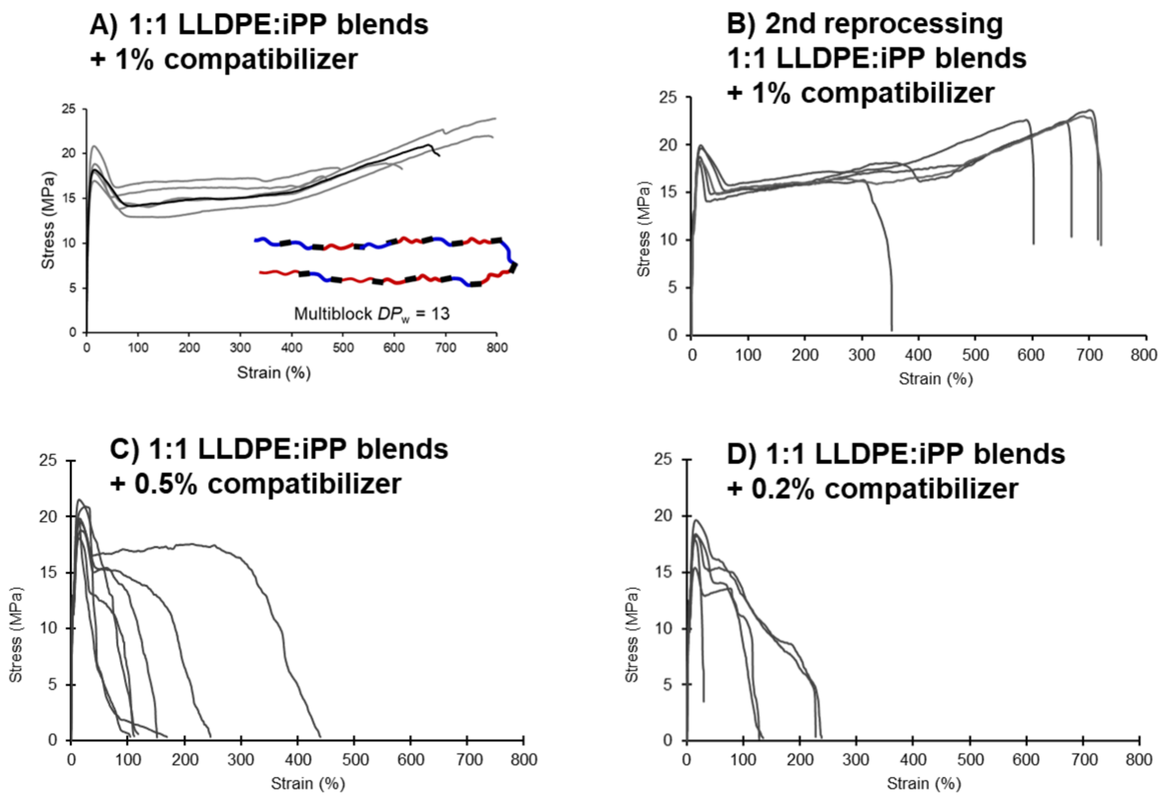

Figure S25. The compatibilization efficiency of $(PE_{12}\text{-}b_{13}\text{-}iPP_{10})_{143}$ in freshly prepared blends (A), after 20 months of aging and subsequent reprocessing at 185 °C for 7 minutes (B), and decreased compatibilizer loading to 0.5 wt% (C) and 0.2 wt% (D) wherein samples are no longer ductile.

Effect of telechelic functionality on degree of polymerization

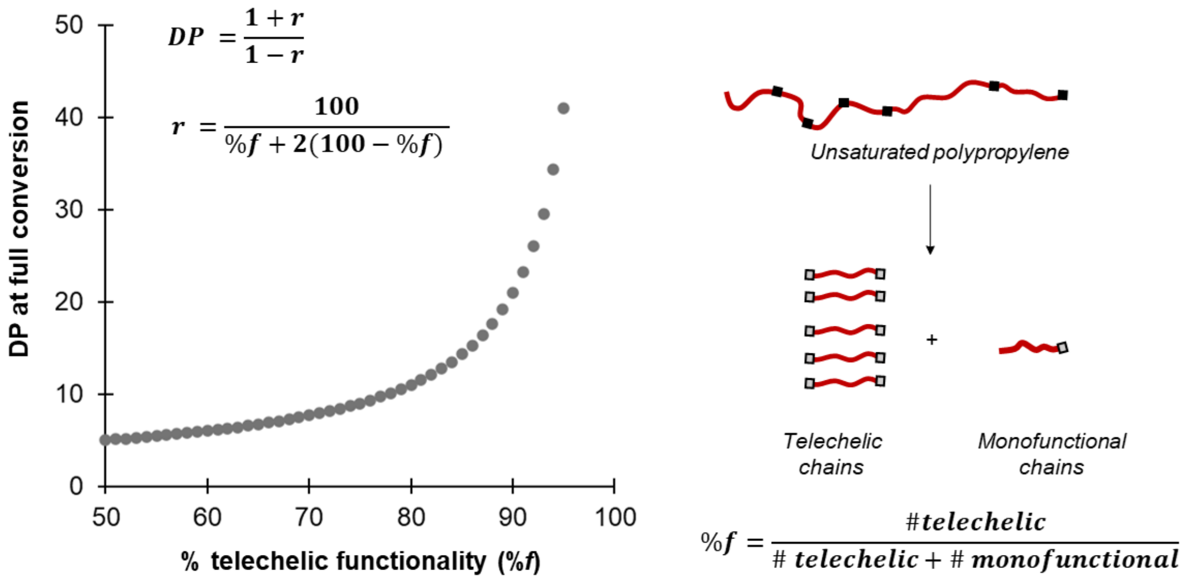

Figure S26. The relationship between maximum degree of shuffling during step growth polymerization and % difunctionality of the telechelic precursors.

Scanning electron microscopy and quantification of average droplet diameter

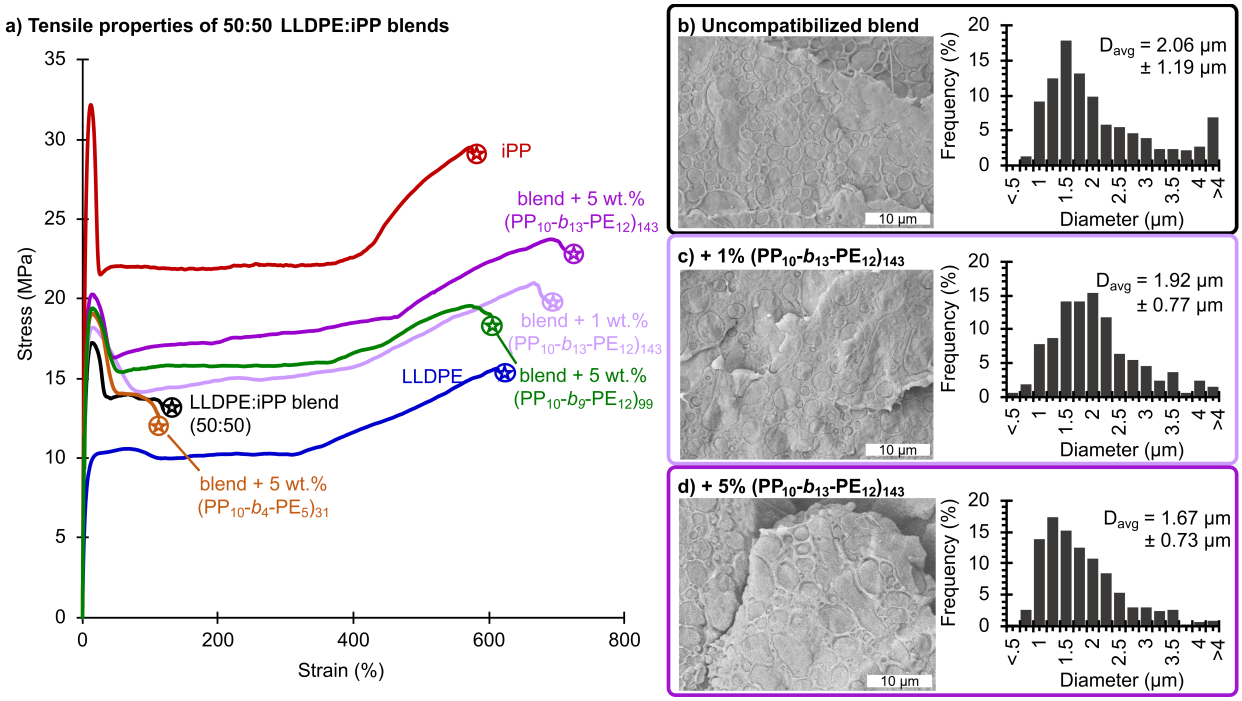

Figure S27. (a) Uniaxial tensile tests at 100%/min of commercial Ziegler-Natta LLDPE, iPP, blends (50:50), and with the addition of multiblock compatibilizers. Scanning electron microscopy and droplet distributions of (b) uncompatibilized blends (c) blends containing 1 wt.% of high molecular weight compatibilizer, and (d) blends containing 5 wt.% of high molecular weight compatibilizer.
